# Introducing a New Algorithm for Classification of Etiology in Studies on Pediatric Pneumonia: Protocol for the Trial of Respiratory Infections in Children for Enhanced Diagnostics Study

**DOI:** 10.2196/12705

**Published:** 2019-04-26

**Authors:** Samuel Arthur Rhedin, Annika Eklundh, Malin Ryd-Rinder, Pontus Naucler, Andreas Mårtensson, Jesper Gantelius, Ingela Zenk, Helene Andersson-Svahn, Susanna Nybond, Reza Rasti, Magnus Lindh, Maria Andersson, Ville Peltola, Matti Waris, Tobias Alfvén

**Affiliations:** 1 Sachs' Children and Youth Hospital South General Hospital Stockholm Sweden; 2 Department of Medical Epidemiology and Biostatistics Karolinska Insitutet Stockholm Sweden; 3 Astrid Lindgren Children's Hospital Stockholm Sweden; 4 Division of Infectious Diseases Department of Medicine, Solna, Karolinska Institutet & Department of Infectious Diseases Karolinska University Hospital Stockholm Sweden; 5 Department of Women's and Children's Health, International Maternal and Child Health (IMCH) Uppsala University Uppsala Sweden; 6 Science for Life Laboratory, Division of Proteomics and Nanobiotechnology KTH Royal Institute of Technology Stockholm Sweden; 7 Department of Public Health Sciences Karolinska Institutet Stockholm Sweden; 8 Department of Infectious Diseases University of Gothenburg Gothenburg Sweden; 9 Department of Paediatrics and Adolescent Medicine Turku University Hospital and University of Turku Turku Finland

**Keywords:** pneumonia, child, preschool, respiratory tract infections, microbiological techniques, diagnostic tests, routine

## Abstract

**Background:**

There is a need to better distinguish viral infections from antibiotic-requiring bacterial infections in children presenting with clinical community-acquired pneumonia (CAP) to assist health care workers in decision making and to improve the rational use of antibiotics.

**Objective:**

The overall aim of the Trial of Respiratory infections in children for ENhanced Diagnostics (TREND) study is to improve the differential diagnosis of bacterial and viral etiologies in children aged below 5 years with clinical CAP, by evaluating myxovirus resistance protein A (MxA) as a biomarker for viral CAP and by evaluating an existing (multianalyte point-of-care antigen detection test system [mariPOC respi] ArcDia International Oy Ltd.) and a potential future point-of-care test for respiratory pathogens.

**Methods:**

Children aged 1 to 59 months with clinical CAP as well as healthy, hospital-based, asymptomatic controls will be included at a pediatric emergency hospital in Stockholm, Sweden. Blood (analyzed for MxA and C-reactive protein) and nasopharyngeal samples (analyzed with real-time polymerase chain reaction as the gold standard and antigen-based mariPOC respi test as well as saved for future analyses of a novel recombinase polymerase amplification–based point-of-care test for respiratory pathogens) will be collected. A newly developed algorithm for the classification of CAP etiology will be used as the reference standard.

**Results:**

A pilot study was performed from June to August 2017. The enrollment of study subjects started in November 2017. Results are expected by the end of 2019.

**Conclusions:**

The findings from the TREND study can be an important step to improve the management of children with clinical CAP.

**International Registered Report Identifier (IRRID):**

DERR1-10.2196/12705

## Introduction

### Biomarkers in Pediatric Respiratory Tract Infections

Respiratory infection is a common reason among children for seeking care [1]. The majority of respiratory infections in children are caused by viruses [2]. Nevertheless, viral and bacterial infections are hard to distinguish clinically, causing many children with viral infections or self-limiting bacterial infections to receive unnecessary antibiotic treatment, which contributes to the development and spread of antibiotic resistance [3,4]. There is a need for new biomarkers that better distinguish viral infections from antibiotic-requiring bacterial infections in children presenting with clinical community-acquired pneumonia (CAP) and that assist health care workers in decision making and improving the rational use of antibiotics [5].

C-reactive protein (CRP), procalcitonin (PCT), and white blood cell (WBC) count are the most commonly used inflammatory markers in clinical practice for the management of children with suspected CAP [6,7]. There is increasing evidence that PCT is superior to CRP as a screening test for serious bacterial infection given the favorable kinetics, including a more rapid response to inflammation [8]. However, neither of the biomarkers have been proven to be reliable in differentiating between mild or moderate bacterial and viral CAP [9,10]. A WBC count of 15,000/μl has been suggested as a cutoff to differentiate between viral and bacterial etiologies. However, critically ill patients with neutropenia will not have an increased WBC count, and certain viruses such as influenza and adenovirus can elicit a strong immune response with a high WBC count greater than 15,000/μl [11]. Neither is a complete blood count reliable in differentiating between bacterial and viral CAP in children [6]. To date, most biomarkers used in clinical practice have been selected for their ability to identify serious bacterial infections, and there is a need for novel biomarkers that can reliably detect *viral* infections [12].

Myxovirus resistance protein A (MxA) is an intracellular protein that is upregulated upon activation of the antiviral defense system. Increased blood MxA has been reported to be specific for viral infection [13-15]. There is a commercially available rapid diagnostic test, FebriDx, that qualitatively detects MxA and CRP at cutoffs of 40 ng/ml and 20 mg/ml, respectively. The test has been reported to have 85% (29/34) sensitivity and 93.4% (183/196) specificity to rule out a bacterial infection in patients (adults and children) with febrile respiratory infection [16]. However, no studies have focused on MxA in children with CAP. It was previously shown that virus-positive asymptomatic children had lower MxA levels as compared with virus-positive symptomatic children with respiratory symptoms [14]. As current viral real-time polymerase chain reaction (PCR) testing of upper respiratory specimens (ie, the routine method for diagnosing respiratory tract infections) is complicated by frequent asymptomatic detection, MxA has the potential to facilitate the interpretation of viral PCR positivity in terms of clinical relevance in children with CAP [17].

### Etiology of Childhood Community-Acquired Pneumonia

Defining etiology in childhood CAP is complex [18]. Until recently, our conception of CAP etiology has largely relied on early lung-aspirate studies from the 1970s to 1980s [19]. Vaccination against *Streptococcus pneumoniae* and *Haemophilus influenzae* type B, the 2 major causative agents in childhood CAP, has been introduced in most parts of the world during the last two decades, coinciding with a global decrease in childhood CAP mortality [20]. This has also contributed to a shift in the etiology of CAP [21,22]. Other important factors include a globally improved socioeconomic and nutritional status, a sharp decrease in the incidence of measles, and the emergence of HIV [5]. Recently, new CAP etiology studies, including the large-scale Pneumonia Etiology Research for Child Health (PERCH) study, have been conducted, mostly with a low-income country focus [21-24]. These have pointed toward an underestimation of viral and mixed viral-bacterial etiologies, which is likely explained by the aforementioned shift in etiology but also by the recent advances in viral diagnostics [25]. Moreover, *Bordetella pertussis* and *Bordetella parapertussis*, the causative agents of whooping cough, have been associated with CAP [21,26]. These bacteria are highly contagious and can cause severe disease, particularly in infants [27]. Recent studies have reported an increasing incidence of *B pertussis*, and there have been several deaths in previously healthy infants associated with whooping cough in Sweden over the last 10 years [28,29]. Consequently, there is a need for new updated studies on CAP etiology in various settings.

### A Need for Rapid Microbiological Point-of-Care Tests

Current treatment options with antivirals for respiratory viruses are limited. However, there are several new antivirals that are being developed [30], and furthermore, there is a value in diagnosing viral infections to predict the clinical course and infectivity and to give confidence to withhold the prescription of antibiotics. Real-time PCR is a sensitive molecular-based method that is currently considered gold standard for the detection of respiratory viruses in children with respiratory tract infection [25]. Nevertheless, as PCR usually has to be run in central laboratories and requires complex instrumentation, the turnaround time can be long and the test results are rarely used for decision making regarding treatment at the point of care. There are currently several new antigen-based point-of-care tests for respiratory infections on the market. One is the multianalyte point-of-care antigen detection test system (mariPOC) respi, ArcDia International Oy Ltd, that uses a 2-photon excitation assay technique to detect 10 different respiratory viruses (influenza A/B, respiratory syncytial virus [RSV], adenovirus, bocavirus, coronavirus, human metapneumovirus [hMPV], and parainfluenza virus [PIV] 1-3) [31]. The advantage of the test is that it gives a preliminary result of strongly positive samples already after 20 min and a final result (including negative results) within 2 hours, which potentially allows for immediate treatment considerations. In children, the mariPOC respi’s sensitivity for RSV and influenza virus has been reported to be as high as 90% as compared with PCR, but the sensitivity for less common respiratory viruses, such as hMPV and PIV, and the newly included coronavirus and bocavirus has been insufficiently investigated [32,33].

Recombinase polymerase amplification (RPA) is a nucleic acid amplification technique that does not require thermal cycling. An RPA-based point-of-care test could combine the advantage of high sensitivity with short turnaround time. An RPA-based test using a paper-based vertical flow microarray technique is currently being developed by our partners at the Science for Life Laboratory (SciLifeLab) [34-37]. As the test reaction is carried out at room temperature, it is an interesting method for resource-limited settings where the need for new diagnostic tests is particularly high [38,39].

### Long-Term Complications of Community-Acquired Pneumonia

Studies on the long-term outcomes of radiologically confirmed bacterial CAP have indicated that the disease is associated with later development of asthma and decreased lung function [40,41]. However, most of these studies have followed children who were born more than two decades ago, and the risk might therefore not generalize to a modern setting, given the reported shift in etiology of pediatric CAP [42]. Hence, there is a need for new studies of long-term complications from pediatric CAP, such as the development of asthma and the risk for future respiratory infections.

In summary, there is a need for (1) assessing the diagnostic accuracy of MxA as a biomarker for viral childhood CAP; (2) new studies on clinical CAP etiology in children; (3) evaluating the antigen-based point-of-care test mariPOC; (4) evaluating a novel RPA-based point-of-care test developed at SciLifeLab; and (V) assessing long-term complications from CAP, including the risk of developing asthma and the risk for future respiratory infections.

The overarching aim of the Trial of Respiratory infections in children for ENhanced Diagnostics (TREND) study is to improve the differential diagnosis of bacterial and viral etiologies in children aged below 5 years with clinical CAP. The specific objectives of the study are as follows:

- the diagnostic accuracy of MxA for viral CAP in children
- etiology of children with CAP
- sensitivity and specificity for the mariPOC respi test for the detection of respiratory viruses
- sensitivity and specificity for a novel RPA-based point-of-care test for the detection of respiratory viruses
- long-term complications in children with CAP.

## Methods

### Study Site and Design

The TREND study is a hospital-based, prospective observational study of children with clinical CAP and asymptomatic controls. The study will take place at Sachs’ Children and Youth Hospital, Stockholm, which has one of the largest pediatric emergency departments in Sweden with over 30,000 visits each year. The study is planned to be conducted from November 2017 to December 2019. The study is registered at clinicaltrials.gov (ID: NCT03233516) July 28, 2017.

### Study Participants

#### Case Definition

**Figure 1:**
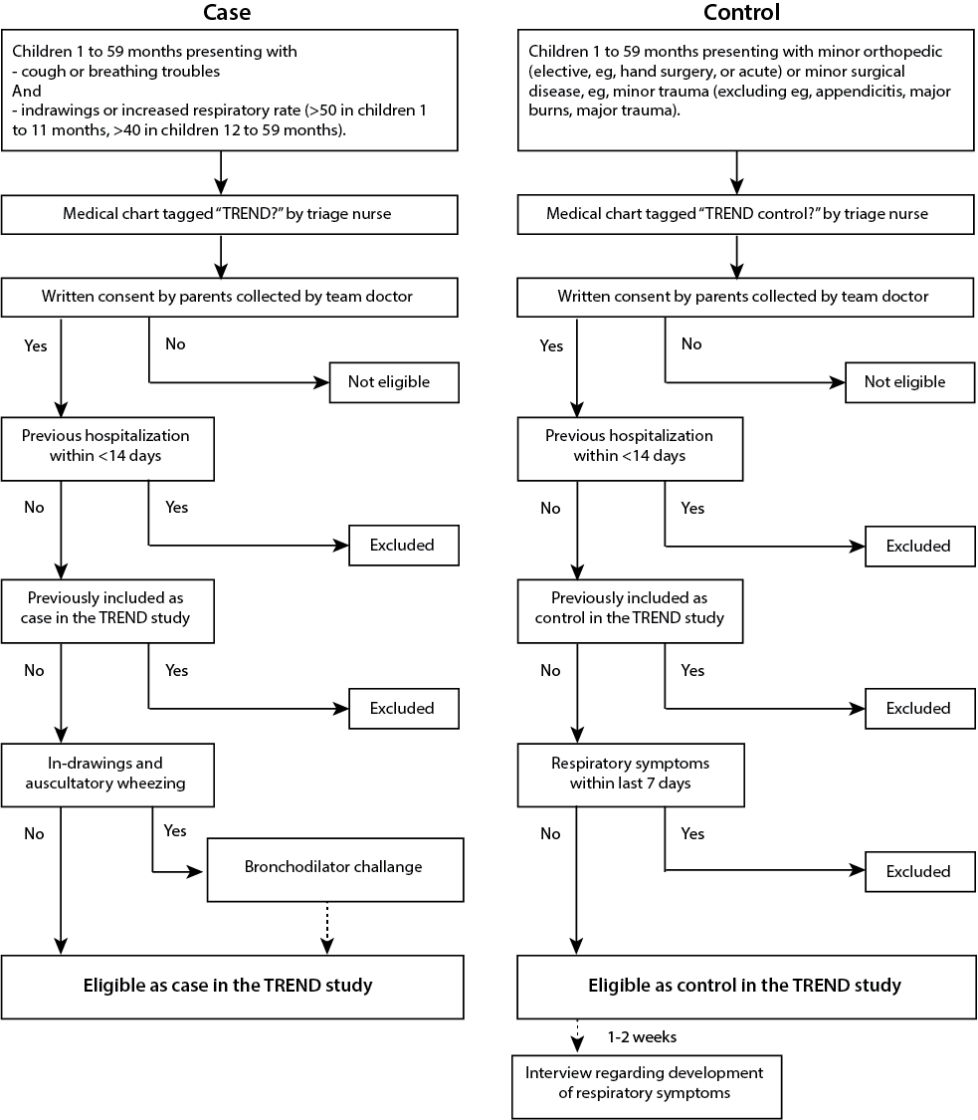
Algorithm for screening and enrollment of study subjects in the Trial of Respiratory infections in children for ENhanced Diagnostics (TREND) study.

Children aged 1 to 59 months with clinical CAP (both severe and nonsevere) according to the World Health Organization (WHO) criteria are enrolled as cases (Figure 1). The inclusion criteria (all inclusion criteria to be met to be eligible for participation in the study) are as follows: age 1 to 59 months, reported or observed breathing troubles/coughing, observed age-adjusted tachypnea (≥50 breaths/min in children aged 1-12 months, ≥40/min in children aged >1 year) or chest indrawing, and written informed consent. The exclusion criteria are as follows: previously included as a case in the study or hospitalized during the previous 14 days. Inhalation with a rapid-acting bronchodilator (≥1 dose in children aged <2 years and ≥3 doses in children aged 2-4 years) will be administered to children with wheezing and chest indrawing to improve the specificity of the WHO clinical CAP criteria, as suggested by the PERCH study team [43]. Resolved chest indrawing after bronchodilator challenge will be recorded but not considered as an exclusion criterion to be able to exclude these patients in a subanalysis as well as to analyze these patients separately.

#### Control Definition

Children aged 1 to 59 months treated for a minor orthopedic (elective, eg, hand surgery, or acute) or minor surgical disease, for example, minor trauma (excluding appendicitis, major burns, major trauma, etc) are enrolled as controls. No formal matching will be performed, but age and season will be considered in the analyses. The exclusion criteria are symptoms of respiratory disease 7 days before enrollment, previous inclusion as a control in the study, or hospitalized during the previous 14 days. The guardians of the controls will be contacted by email/telephone 1 to 2 weeks after enrollment to collect information regarding the potential respiratory symptoms developed after discharge. Cases and controls will be included in the emergency unit, and additional controls will also be included in the hand surgery unit of the hospital.

### Biological Samples

Capillary blood samples and nasopharyngeal aspirates and swabs will be collected from all study subjects. For the MxA analysis, 20 µl of blood will be collected using a heparinized plastic end-to-end capillary and then immediately diluted in a prefilled tube containing an in-house buffer [14]. The nasopharyngeal swabs for the mariPOC respi analyses will be diluted in 1.3 ml of a commercial buffer, as advised by the manufacturer ArcDia International Oy Ltd. The nasopharyngeal aspirates for the PCR analyses will be collected in a standardized manner, as has previously been published, but mixed with 1.3 ml of saline to mirror the protocol for the mariPOC respi [17]. All samples will be taken within 24 hours from arrival at the emergency unit, and the time of blood and nasopharynx sampling will be recorded. It will also be noted if antibiotics have been given before sampling. Samples that are not analyzed at the point of care will be stored at −80°C and shipped on dry ice to minimize degradation of the analytes of interest. All samples collected in the study will be stored according to the Swedish act: Biobanks in Medical Care (SFS 2002:297).

### Microbiological and Biochemical Analyses

Real-time PCR analysis based on the TaqMan technique will be performed in batches on the nasopharyngeal aspirates at Sahlgrenska University Laboratory, Gothenburg, using previously described methods [44]. The PCR detects the following respiratory agents: influenza A/B, RSV A/B, adenovirus, bocavirus, coronavirus (HKU1, NL63, OC43, and 229E), hMPV, PIV 1-3, rhinovirus, enterovirus, *S pneumoniae*, *H influenzae,* *B pertussis*, and *Mycoplasma pneumoniae*.

mariPOC respi will be performed on the nasopharyngeal swabs directly at the emergency room at Sachs’ Children and Youth Hospital at the time of enrollment [32]. The test detects the following respiratory agents: influenza A/B, RSV, adenovirus, bocavirus, coronavirus, hMPV, PIV 1-3, and *S pneumoniae.*

Analyses of MxA will be performed in batches at the Institute of Biomedicine, University of Turku, Finland, using an in-house enzyme immunoassay, as previously described [14].

CRP will be analyzed using the Alere Afinion AS100 Analyzer commercial point-of-care kit at the emergency room at Sachs’ Children and Youth Hospital. If multiple CRP tests are performed, the highest value less than 48 hours from arrival at the emergency unit will be recorded [45]. A small amount of blood will be stored to allow future analysis of, for example, PCT if deemed necessary.

### Study Variables

Information regarding the study subjects (initials, year and month of birth, sex, date of inclusion, and postal code), number of siblings, days of illness, current symptoms (fever, coughing, runny nose, wheezing, whooping, shortness of breath, hoarseness, sore throat, ear secretion, inability to feed, lethargy, vomiting, and diarrhea), vaccinations, antibiotic treatment, medication, underlying diseases, heredity for asthma, previous hospitalization, recent (last 3 months) trips abroad (if yes, where and for how long), allergies, smoking in the family, recent contact with unwell individuals, breastfeeding, preschool, origin of parents, and socioeconomic status will be collected through a standardized questionnaire based on previous studies [46,47].

Clinical parameters (respiratory rate, consciousness according to the AVPU scale—alert, verbal stimuli, pain stimuli, unresponsive—pulse, peripheral oxygen saturation, weight, body temperature, vomiting, head nodding, central cyanosis, stridor, chest indrawing, nasal flaring, grunting, pedal edema, skin turgor, capillary refill, cool peripheries, and pulmonary auscultatory findings—decreased breath sounds, crackles/crepitations, bronchial breath sounds, and wheezing) and antipyretic medication prescribed within less than 4 hours will be registered by the study doctor responsible for patient screening/enrollment. To avoid overloading the case report form, information about symptoms and danger signs that are rare in a Swedish context (eg, jaundice, bulging fontanelle, rash, gallop rhythm, weak peripheral pulses, and tender liver mass) will be retrospectively collected from the medical records if deemed necessary. Some clinical parameters are routinely recorded multiple times at the emergency unit. In these cases, the most extreme value (highest pulse/respiratory rate/body temperature and lowest peripheral oxygen saturation) during the visit at the emergency unit enrollment will be recorded. Information regarding admission; length of hospital stay; routine clinical examination; radiological, microbiological, and biochemical analyses (eg, bacterial cultures and blood gas tests); treatment; discharge diagnosis; and complications (parapneumonic effusion and sepsis) will be retrospectively collected from the medical records.

Personal identification numbers of all the study subjects will be linked to the national health and population registers to collect information regarding deaths, previous immunization, and discharge diagnoses according to the International Statistical Classification of Diseases and Related Health Problems 10 (ICD-10) as well as Anatomic Therapeutic Chemical classification system codes for prescribed drugs.

### Classification of Etiology in the Trial of Respiratory Infections in Children for Enhanced Diagnostics Study

**Figure 2:**
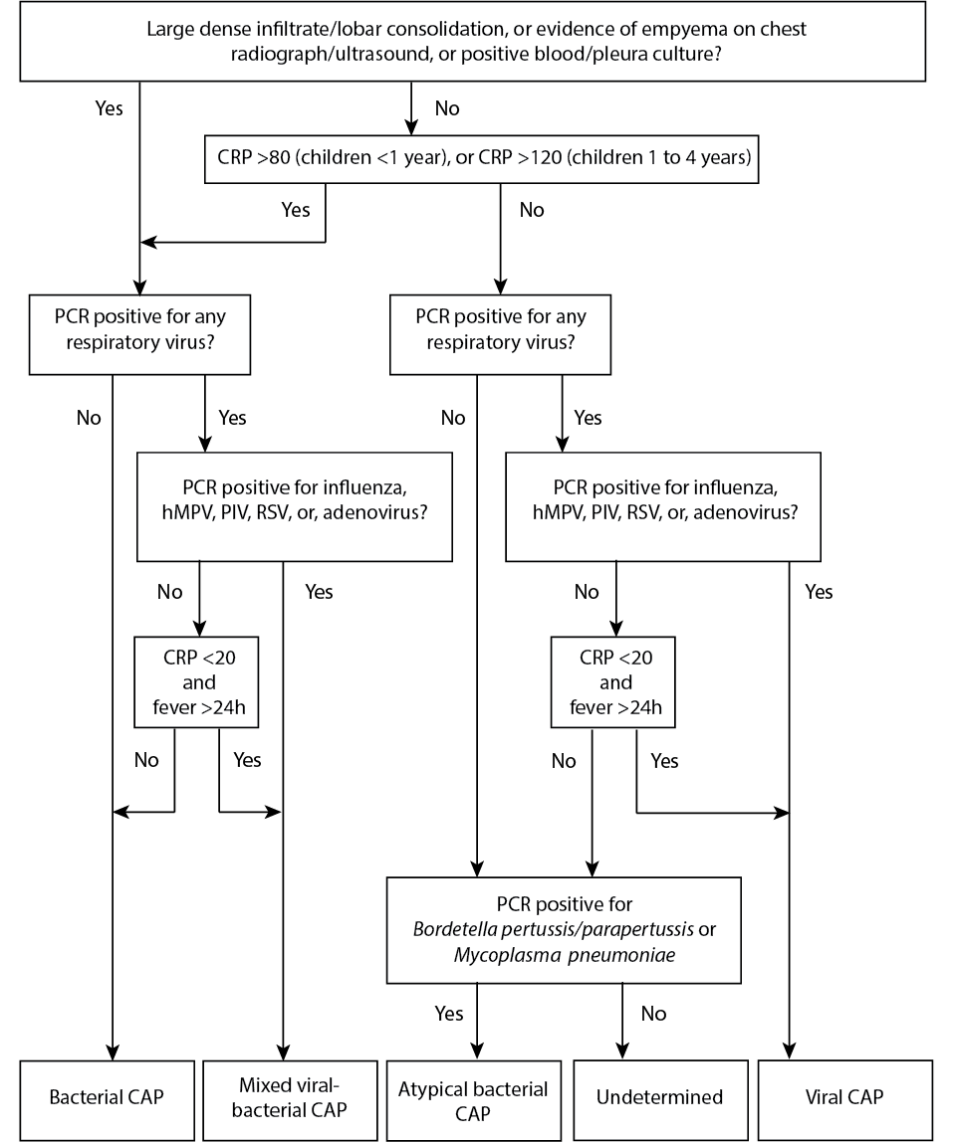
Classification of community-acquired pneumonia etiology in the Trial of Respiratory infections in children for ENhanced Diagnostics (TREND) study. CAP: community-acquired pneumonia; CRP: C-reactive protein; hMPV: human metapneumovirus; PCR: polymerase chain reaction; PIV: parainfluenza virus; RSV: respiratory syncytial virus.

**Figure 3:**
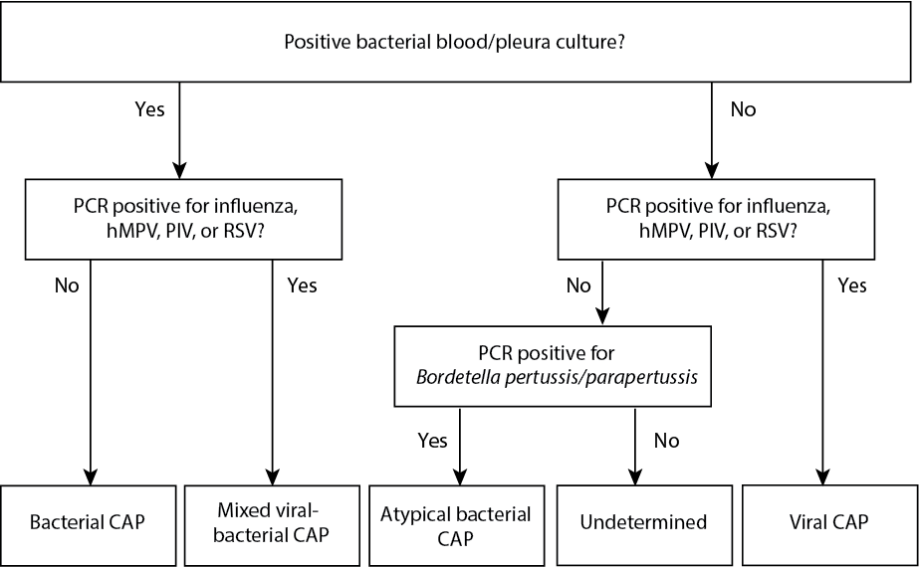
Classification of community-acquired pneumonia (CAP) etiology in the Trial of Respiratory infections in children for ENhanced Diagnostics study—strict definition. CRP: C-reactive protein; hMPV: human metapneumovirus; PCR: polymerase chain reaction; PIV: parainfluenza virus; RSV: respiratory syncytial virus.

The algorithm for classifying etiology in the TREND study is based on the current literature and will classify children into viral, bacterial, atypical bacterial, mixed viral-bacterial, and undetermined infections based on clinical, microbiological, radiographic, and biochemical findings (Figure 2). A second, stricter algorithm only considering microbiologically confirmed diagnoses will be used in a complementary subanalysis (Figure 3).

### Long-Term Complications

Long-term complications (asthma and number of hospital-requiring respiratory infections) will be assessed by linking to the National Patient Register. Asthma will be classified according to the ICD-10 diagnosis and/or prescriptions of asthma medication in the Swedish Prescribed Drug Register using a previously validated algorithm [48].

### Study Size and Power Calculation

A total of 300 cases and 120 controls are estimated to be included in the TREND study. For the sample size calculation, we focused on the assessment of MxA levels in cases with viral CAP as compared with cases with bacterial CAP/controls (study I). Overall, 2 power calculations were made, 1 for viral CAP versus bacterial CAP and 1 for viral CAP versus controls. The following assumptions were made: (1) A difference in MxA level of 500 µg/l between the groups was considered clinically relevant. (2) A standard deviation of 1000 and 300 was assumed in cases with viral CAP and bacterial CAP/controls, respectively, based on previous studies on MxA [13,14]. Using an alpha level of .05 (2 sided) at an 80% power, with an additional 20% to account for nonparametric testing and multivariate analyses, 42 children in each group (viral CAP, bacterial CAP, and controls) would be needed. To ensure that enough of the included cases would fulfill the study definitions for viral and bacterial CAP, the proportion of children with viral and bacterial CAP (TREND definition) was calculated in our previous study that assessed Swedish children with x-ray–verified CAP [47]. By doing this, the prevalence of viral and bacterial CAP was estimated at 45% and 14%, respectively. Hence, 300 cases and 42 controls would be needed to ensure sufficient collection of cases with viral and bacterial CAP, respectively. We also would like to compare viral CAP cases with controls testing positive for 1 or more viruses by PCR. In our previous study, 35.4% of asymptomatic children tested positive for 1 or more viruses. Hence, to include a sufficient number of virus-positive controls, we aim at including 300 cases and 120 controls in the TREND study.

### Statistical Methods

A clinically relevant difference in MxA levels will be compared between cases with viral and bacterial clinical CAP as well as between cases with viral clinical CAP and controls using appropriate statistical methods according to the number and distribution of data points. Sensitivity and specificity for different respiratory viruses with mariPOC respi and the novel RPA-based test will be calculated as compared with real-time PCR. The difference in asthma prevalence and the difference in the number of hospital-requiring respiratory infections between cases and controls as well as between cases and the general child population will be assessed after 3, 7, and (if deemed necessary) 10 years. Data will be presented with 95% CI, and a *P* value of <.05 will be considered significant.

### Ethics Approval and Consent to Participate

The study will be conducted in accordance with the latest version of The Declaration of Helsinki and the fundamental principles of respect for the individual’s (Article 8) right to self-determination and to make informed decisions (Articles 20, 21, and 22) regarding participation in research, both initially and during the course of the research.

We estimate that the benefit of knowing more about viral respiratory infections with the aim of improving diagnostics of CAP overweighs the discomfort for the individual study participant in terms of extra sampling. For all the participating children, a minimal, reduced amount of blood will be collected, and accordingly, the analysis of PCT and CRP will not routinely be performed in control children for the following reasons: (1) data on these biomarkers in the controls are not necessary for the study objectives; (2) to get a sufficient amount of blood for running these analyses, it would require a larger lancet and/or additional punctures; and (3) these children would likely not have been subject to capillary puncture, were they not enrolled in the study. Results from point-of-care tests will be provided to guardians and treating physicians. Other test results will not be provided as they will be analyzed in batches and thus not influence management. Written informed consents will be collected from the guardian(s) by the study nurse/physician before sampling. To ensure confidentiality for the participants, samples will be given a study ID and results will only be presented at a group level. Data of personal identities will be stored in a password-protected data file at Sachs’ Children and Youth Hospital and will only be available to the study researchers. Good clinical practice and good laboratory practice will be followed. The study was approved by the Regional Ethical Review Board in Stockholm (ref 2017/958-31).

## Results

The study was approved by the Regional Ethical Review Board in Stockholm in June 2017 (ref 2017/958-31). A pilot study was performed from June to August 2017 to evaluate the study protocol from a logistical and methodological point of view. Overall, 6 out of 9 invited cases and 1 out of 3 invited controls were included. Valuable information was retrieved during the pilot study, which has led to alterations and improvements in the recruitment process, the questionnaire, and other study documents as well in the logistics and handling process of the samples. Enrollment of study subjects started in November 2017. Results are expected by the end of 2019.

## Discussion

### Principal Findings

The TREND study aims to improve the differential diagnosis of bacterial and viral etiology in children aged below 5 years with clinical CAP presenting at an emergency unit in a tertiary pediatric hospital in Sweden. This is the first study of children with clinical CAP that evaluates the diagnostic accuracy of MxA as a biomarker for viral CAP. Previous studies on MxA have shown promising results on the role of MxA as a biomarker for viral infection but have been smaller in size [14] or have included more heterogeneous groups of study subjects [13,15,16]. The TREND study aims to add further information on the role of MxA as a marker to improve the interpretation of viral PCR positivity and to differentiate between viral and bacterial infections with a specific focus on children with CAP. Further aims are to validate the findings from recent pediatric CAP etiology studies, including the EPIC and PERCH studies, as well as to assess the long-term complications of pediatric CAP [21-24].

### A New Algorithm for Classification of Etiology in Pediatric Community-Acquired Pneumonia Studies

One major weakness in the studies of diagnostic biomarkers in pediatric infectious diseases is the lack of a reliable gold standard for the microbiological diagnosis [5,49]. The gold standard for assigning bacterial etiology has traditionally been the detection of bacteria in cultures from normally sterile sites (lung, blood, and pleura). However, as sampling from the lung/pleura is infeasible in most cases and blood cultures have limited sensitivity, this approach is of little use in clinical studies of CAP [5]. Previous studies assessing the performance of diagnostic tests in terms of distinguishing bacterial infections from viral infections have used either an independent expert panel or a laboratory-/radiological-based approach to classify disease etiology [12-15]. Both approaches have their advantages and limitations. Using a strict microbiologically confirmed diagnosis as the reference has the advantage of high specificity, but the generalizability of the findings is hampered as the majority of children in clinical practice will not have a clear microbiologically confirmed diagnosis. In addition, for a diagnostic test to be useful, it is more important to distinguish between etiologically less clear cases of respiratory infections rather than to identify the school book examples of bacterial and viral infections. In the TREND study, we chose a more pragmatic approach favoring generalizability for microbiological accuracy. However, given that doctors in expert panels rely on microbiological and biochemical findings, we reasoned that it still would be more stringent to create an algorithm for the classification of etiology. In the TREND study, a diagnostic algorithm has been created a priori to serve as the reference based on the current evidence for the classification of CAP etiology. Detection of RSV, hMPV, influenza virus, and PIV has, in previous case-control studies, been highly associated with CAP. Furthermore, these viruses appear to be rarely detected in asymptomatic individuals, and hence, detection will be considered to be a definitive indicator of etiology [21,22,47,50,51]. For other respiratory viruses as well as for the atypical bacteria *M pneumoniae*, the clinical significance of PCR positivity is less clear owing to frequent detections in asymptomatic children [17,52-55]. For that reason, PCR positivity will not be considered enough for establishing etiology. Virkki et al reported that a CRP level of more than 80 in children aged less than or equal to 2 years and more than 120 in children aged 2 to 5 years was specific (>85%) for bacterial etiology, whereas a CRP level of less than 20 was specific for viral etiology (78%) [56]. These cutoffs will be used to aid in the definition of probable bacterial and viral infections in less clear cases. Hence, detection of viruses other than influenza, RSV, hMPV, and PIV will be considered as viral infections only if the CRP value is less than 20. Given that adenovirus has been associated with high CRP, this rule will not be applicable for adenovirus [11]. However, adenovirus detections will not be considered in the strict algorithm, as asymptomatic detection of the virus is common [17]. Finally, given that CRP levels depend on the disease duration and to avoid false-negative test results (ie, low but rising CRP values), an additional criterion of reported fever duration of more than 24 hours will be applied in the TREND study when considering CRP levels [45]. As discussed above, there is no optimal reference standard for the classification of CAP etiology, neither clinically nor in research. However, we believe that much is to be gained if we use an algorithm instead of an expert panel. The decisions taken by expert panels differ between different studies, over time, and between different settings. When using an algorithm, this can be controlled for. We look forward to comments and inputs on our algorithm so that together we can develop it further.

### Difficulties in Pediatric Community-Acquired Pneumonia Etiology Studies

The WHO criteria for clinical CAP lack specificity and will result in the inclusion of a significant proportion of children who will not have true CAP, including children with bronchiolitis and asthma [57]. To improve the specificity of the WHO criteria for clinical CAP, a rapid-acting bronchodilator will be administered to children with wheezing and chest indrawing, as suggested by the PERCH study team [43]. Other clinical parameters from the PERCH study will also be included and used for further subanalyses [43].

Conducting research in the pediatric emergency department is difficult [58]. High patient flows and long waiting times create a stressful environment for all personnel categories. Motivating nurses/doctors to spend the extra time and effort it takes to recruit patients is, therefore, challenging. Continuous education/information about the research project, interpersonnel teamwork (nurse and doctor), and incentives are all key success factors. Recruitment of healthy controls in this age group is an obvious challenge as the sampling (blood and nasopharyngeal sampling) causes discomfort to the child. Therefore, attempts have been made to include patients who will undergo elective hand surgery (and thereby be sedated during the sampling). Certain clinical parameters can be deceptive if not recorded correctly, which is a potential source of bias. In children where peripheral oxygen saturation and heart rate are measured continuously, data will still only be recorded at certain time points when the children are at rest.

### Requirements of Future Rapid Diagnostic Tests

Transcriptomic studies have shown promise in differentiation between different infectious agents but currently require advanced instrumentation with a long turnaround time and are hence more suitable for an intensive care unit setting than for routine testing at pediatric emergency units [59]. However, given the complexity of the host immune response elicited by respiratory pathogens, it is possible that a single biomarker will not be sufficient to accurately differentiate between viral and bacterial CAP. However, MxA could be valuable in a rapid combination test of biomarkers and selected microbiological testing if it proves to be specific for viral CAP. Such commercial combination point-of-care tests of inflammatory biomarkers are already being developed and some, such as FebriDx and MeMed BV, have shown promise [15,60].

Improved near-patient differential diagnosis is a prerequisite for rational antibiotic use and decreasing unnecessary antibiotic treatment. Furthermore, easier identification of the pathogens causing acute respiratory infections makes it easier to advise guardians to care for their sick children and for better disease surveillance in the society. Hence, the findings from the TREND project can be an important step toward the improved care of children with clinical CAP. At this stage, the methods are developed and evaluated in a Swedish context but might have wider implications, for example, to resource-limited settings where the need for similar tests is even higher than in a high-income context such as Sweden.

## Abbreviations

CAP: community-acquired pneumonia
CRP: C-reactive protein
hMPV: human metapneumovirus
ICD-10: International Statistical Classification of Diseases and Related Health Problems 10
mariPOC: multianalyte point-of-care antigen detection test system
MxA: myxovirus resistance protein A
PIV: parainfluenza virus
PCR: polymerase chain reaction
PCT: procalcitonin
PERCH: Pneumonia Etiology Research for Child Health
RPA: recombinase polymerase amplification
RSV: respiratory syncytial virus
TREND: Trial of Respiratory infections in children for ENhanced Diagnostics
WBC: white blood cell
WHO: World Health Organization
